# Magnetopriming Actuates Nitric Oxide Synthesis to Regulate Phytohormones for Improving Germination of Soybean Seeds under Salt Stress

**DOI:** 10.3390/cells11142174

**Published:** 2022-07-12

**Authors:** Sunita Kataria, Anjali Anand, Ritesh Kumar Raipuria, Sunil Kumar, Meeta Jain, Anshul Watts, Marian Brestic

**Affiliations:** 1 School of Biochemistry, Devi Ahilya Vishwavidyalaya, Khandwa Road, Indore 452001, India; meetajainind@yahoo.com; 2 Division of Plant Physiology, ICAR-Indian Agricultural Research Institute, New Delhi 110012, India; 3 ICAR-National Institute for Plant Biotechnology, New Delhi 110012, India; raipuriaritesh@gmail.com (R.K.R.); anshiari@gmail.com (A.W.); 4 Division of Seed Science and Technology, ICAR-Indian Agricultural Research Institute, New Delhi 110012, India; sspatil1550@gmail.com; 5 Institute of Plant and Environmental Sciences, Slovak University of Agriculture, A. Hlinku 2, 94976 Nitra, Slovakia; marian.brestic@gmail.com; 6 Department of Botany and Plant Physiology, Faculty of Agrobiology, Food and Natural Resources, Czech University of Life Sciences Prague, 16500 Prague, Czech Republic

**Keywords:** germination, growth hormones, magnetopriming, nitric oxide, salt stress, tolerance

## Abstract

In this study, the role of the signalling molecule nitric oxide (NO) in magnetopriming-mediated induction of salinity tolerance in soybean seeds is established. The cross-talk of NO with germination-related hormones gibberellic acid (GA), abscisic acid (ABA) and auxin (IAA) for their ability to reduce the Na^+^/K^+^ ratio in the seeds germinating under salinity is highlighted. Salt tolerance index was significantly high for seedlings emerging from magnetoprimed seeds and sodium nitroprusside (SNP, NO-donor) treatment. The NO and superoxide (O_2_^•−^) levels were also increased in both of these treatments under non-saline and saline conditions. NO generation through nitrate reductase (NR) and nitric oxide synthase-like (NOS-like) pathways indicated the major contribution of NO from the NR-catalysed reaction. The relative expression of genes involved in the NO biosynthetic pathways reiterated the indulgence of NR in NO in magnetoprimed seeds, as a 3.86-fold increase in expression was observed over unprimed seeds under salinity. A 23.26-fold increase in relative expression of NR genes by the NO donor (SNP) was observed under salinity, while the NR inhibitor (sodium tungstate, ST) caused maximum reduction in expression of NR genes as compared to other inhibitors [L-NAME (N(G)-nitro-L-arginine methyl ester; inhibitor of nitric oxide synthase-like enzyme) and DPI (diphenylene iodonium; NADPH oxidase inhibitor)]. The ratio of ABA/GA and IAA/GA decreased in magnetoprimed and NO donor-treated seeds, suggesting homeostasis amongst hormones during germination under salinity. The magnetoprimed seeds showed low Na^+^/K^+^ ratio in all treatments irrespective of NO inhibitors. Altogether, our results indicate that a balance of ABA, GA and IAA is maintained by the signalling molecule NO in magnetoprimed seeds which lowers the Na^+^/K^+^ ratio to offset the adverse effects of salinity in soybean seeds.

## 1. Introduction

Seed germination is the most crucial stage for crop establishment, especially under soil-related stresses such as salinity, drought, nutrient deficiency, heavy metals, etc. Amongst these, soil salinity is one of the major abiotic stresses which is detrimental in achieving a uniform and healthy crop stand in many crop plants [1,2]. It hampers the overall plant growth by delaying seed germination and reducing germination rate, to finally retard the optimum growth of seedlings [3]. Salt stress causes several physiological and biochemical abnormalities such as ionic toxicity, osmotic imbalance and mineral deficiency due to high Na^+^ influx [4]. The menace of soil salinity is getting worse with time, and if left unchecked will turn 50% of the world’s arable land into salt-affected soils by 2050 [2,3]. In addition to restricting salinization and investing in reclamation of degraded saline soils, seed/plant-based approaches must be adopted for integrated management of the impending crisis.

Soybean (*Glycine max* (L.) Merrill) is an economically important legume crop cultivated around the world, principally for vegetable oil and protein feed for humans as well as livestock [5]. However, the germination of soybean and its overall performance is severely affected by salt stress [3,6]. Hence, it becomes pertinent to examine the potential of seed enhancement techniques in lowering the risk involved in achieving a reasonably good plant population under the stressed environment. Seed priming is a technique to stimulate seed metabolic processes during germination and improve their performance under diverse environmental conditions [7,8]. Magnetopriming is a non-invasive dry seed priming treatment that has been widely used to enhance seedling vigor and plant growth under various environmental cues [9,10,11,12,13]. It can improve seedling growth under various abiotic stresses (drought, salt, heavy metal toxicity and UV-B stress) via increased metabolic processes like α-amylase, protease, nitrate reductase and nitric oxide synthase-like activities during germination [9,12,14,15,16].

The biological effects of magnetopriming may be explained as an interaction of magnetic field and ionic current in the plant embryo cell membrane that leads to changes in both ionic concentrations and osmotic pressure on both sides of the membrane [17]. Changes in water uptake mechanisms are caused by alterations in the ionic fluxes across the cell membrane [18]. During seed germination, the faster imbibition in magnetoprimed seeds lead to early hydration of macromolecules and higher activities of enzymes such as α-amylase and nitrate reductase that are responsible for faster germination of magnetoprimed seeds [15,16].

Nitric oxide (NO) and reactive oxygen species (ROS) are the apparent signalling molecules that play a crucial role in regulating seed dormancy and germination [9,12,13,14,15]. Apart from various reports on ROS, evidence on NO-based regulation of seed germination, photosynthesis, flowering, senescence, stomatal movement, stress tolerance, ROS scavenging and defence mechanisms in different crop species is also accumulating [16,19,20,21,22,23,24,25]. However, it still remains an intriguing subject for the scientific community, as both NO and ROS serve as secondary messengers to regulate several biological processes. In our recent studies, we also provided evidence on the involvement of NO in conferring stress tolerance, through its production in nitrate reductase and nitric oxide synthase-like catalysed reactions in magnetoprimed soybean seeds under salt and UV-B stress [15,16,26]. 

The signalling role of NO and ROS is coordinated through a network of hormones that regulate the germination process. Gibberellic acid (GA) and abscisic acid (ABA) have been unequivocally established in breaking seed dormancy and promoting seed germination by their antagonistic action [27,28,29]. The ratio of ABA/GA is a decisive factor in the maintenance or breaking of dormancy in the seeds [7,30]. The response of germination to the endogenous auxins also takes place in an ABA-dependent manner [31,32]. The source of free auxins in the germinating seeds is the indole acetic acid (IAA) conjugates that are accumulated during seed development and maturation [33]. Studies on the interaction of NO and ROS (O_2_^•−^) with ABA, GA and IAA to regulate the salt tolerance in magnetoprimed germinating soybean seeds would be interesting to delineate the signalling role of the two signalling molecules with the phytohormones. Keeping this in view, we investigated the role of enzymes, nitric oxide synthase-like (NOS-like) as oxidative and nitrate reductase (NR) as reductive pathway towards NO production and its interaction with growth hormones ABA, GA and IAA to confer salt tolerance by maintaining reduced levels of Na^+^/K^+^ ratio in germinating magnetoprimed soybean seeds. For this, we used a NO donor (sodium nitroprusside, SNP), inhibitors of NR (sodium tungstate, ST), or NOS-like enzyme (N(G)-nitro-L-arginine methyl ester, L-NAME) and an inhibitor of superoxide production by the enzyme NADPH oxidase (diphenylene iodonium, DPI).

## 2. Material and Methods

The soybean (*Glycine max* (L.) Merrill) var. JS-335 seeds were procured from ICAR-Indian Institute of Soybean Research, Khandwa Road, Indore, Madhya Pradesh, India. 

### 2.1. Seed Priming with Static Magnetic Field

Soybean seeds were subjected to magnetopriming by placing the seeds under astatic magnetic field (SMF) of 200 mT strength for 1 h according to the previously standardized method of treatment for soybean seeds [15,34]. Testron EM-20 (Testron Instruments, Delhi, India), an electromagnetic field generator with adjustable horizontal magnetic field strength (50–500 mT), was used for seed treatment. The pole pieces were cylindrical with 9 cm diameter and 16 cm length with a gap of 5 cm between the two pole pieces. There were 3000 turns per coil and the resistance of the coil was 16 Ω. For the electromagnet, a direct current (DC) power supply (80 V/10 A) through continuous variable output current was used. The field strength produced in the pole was monitored through a digital Gauss meter model DGM-30 (Testron Instruments, Delhi, India). The probe is made up of Indium Arsenide crystal encapsulated by a non-magnetic sheet of 5 × 4 × 1 mm and can measure 0–2 T with full scale range in increments of 5 mT. 

After magnetopriming treatment, the seeds were surface sterilized by rinsing them in 0.01% HgCl_2_ for 2 min followed by washing 5–6 times with distilled water and drying between layers of blotting paper. 

### 2.2. Seed Germination under Saline and Non-Saline Conditions

The control (unprimed-UP) and magnetoprimed (static magnetic field strength of 200 mT for 1 h-MP) seeds were kept for germination in Petri plates (15 cm × 2 cm) lined with Whatman filter paper no. 1 soaked in 10 mL of various modulators of NO and ROS synthesis, under non-saline (distilled water) and saline (50 mM NaCl) conditions. The NO and ROS modulators included 200 μM sodium nitroprusside (SNP; NO donor), 200 μM of N(G)-nitro-L-arginine methyl ester (L-NAME; inhibitor of nitric oxide synthase-like enzyme), 2000 μM of sodium tungstate (ST; nitrate reductase inhibitor) and 100 μM of diphenylene iodonium (DPI; NADPH oxidase inhibitor) with and without 50 mM NaCl solution, as previously described by [15] (Table 1). For the analysis of biochemical/molecular parameters, the Petri plates were kept for 24 h and for salt tolerance index (dry weight of seedlings) and Na^+^ and K^+^ concentrations for 72 h in an incubator at 25 ± 1 °C in complete darkness.

### 2.3. Salt Tolerance Index (STI)

Salt tolerance index of 72 h germinated unprimed and magnetoprimed soybean seeds under non-saline and saline conditions were calculated by the following formula given by Goudarzi and Pakniyat [35].

$$\mathrm{Salt}\;\mathrm{Tolerance}\;\mathrm{Index}=\frac{\mathrm{Dry}\;\mathrm{weight}\;\mathrm{of}\;\mathrm{seedlings}\;\mathrm{under}\;\mathrm{saline}\;\mathrm{conditions}}{\mathrm{Dry}\;\mathrm{weight}\;\mathrm{of}\;\mathrm{seedlings}\;\mathrm{under}\;\mathrm{nonsaline}\;\mathrm{conditions}}$$

### 2.4. In-Vivo Measurement of Na^+^ and K^+^ Ions

Soybean seedlings (200 mg) from 72 h germinated seeds were oven dried at 80 °C till constant dry weight was obtained. The dried seedlings were powdered finely and digested overnight with 10 mL of diacid solution (HNO_3_:HCLO_4_; 9:4). The beakers containing digested material were kept for further digestion at 278 °C for 1–2 h until a clear solution was obtained. The solution was transferred to volumetric flasks and the volume was increased to 25 mL with double distilled water. The resultant solution was used for the analysis of K^+^ and Na^+^ using a flame photometer [36].

### 2.5. Estimation of Endogenous ABA, GA and IAA Levels

The phytohormones in the 24 h soaked seeds from different treatments of NO and ROS modulators were assessed by following the procedure of Kelen et al. [37] to record any change before the radicle protrusion. The 24 h soaked seeds (1 g) were homogenized in 10 mL of cold Na-phosphate buffer (0.05 M, pH 7.5) containing antioxidant (0.02% sodium diethyldithiocarbamate). The homogenate was kept overnight at 4 °C in a shaker at 150 rpm. The samples were centrifuged at 10,000 rpm for 15 min at 4 °C and the supernatant was separated. The samples were partitioned in a separating funnel using 5 mL diethyl ether and the pH of resultant aqueous phase was adjusted to 2.5 using 1 N HCl, followed by further partitioning twice with 5 mL petroleum ether. The ether phase from the previous step was discarded and aqueous phase was further partitioned thrice with 5 mL diethyl ether. The ether phase emanating from this step was filtered with sodium sulphate crystals using Whatman no. 1 filter paper. The filtrate was lyophilized and resolved in 100 µL of mobile phase consisting of acetonitrile (26% *v*/*v*) for identification of ABA, GA and IAA. The extract was filtered through a 0.22 mm syringe filter, collected in HPLC vials and stored at 4 °C temperature for further usage. Analysis was carried out on an Agilent 1100 series HPLC value system. The separation was accomplished by a 4.6 mm × 250 mm stainless steel analytical column packed with 5 µm particle size (Zorbax eclipse XDB C-18, Agilent). The lyophilized sample was dissolved in acetonitrile:water (26:74) and analysed in an Agilent zorbax eclipse XDB C-18 column (4.6 mm × 250 mm Agilent Technologies 1100/1200 Series, Santa Clara, CA, USA) maintained at 35 °C with the UV-Visible detector with a flow rate of 0.8 mL/min. The wavelengths used for the determination of ABA, GA and IAA were 206, 265 and 280 nm, respectively. The standards of the three hormones at varying concentrations were also included with each group of samples loaded onto the HPLC.

### 2.6. Estimation of Superoxide (O_2_^•−^) and Nitric Oxide (NO) Content

Superoxide (O_2_^•−^) content in each sample was determined by the method of Chaitanya and Naithani [38]. One hundred milligram of soybean (24 h soaked) seeds from different treatments were homogenised with chilled sodium phosphate buffer (0.2 M; pH 7.2) containing diethyldithiocarbamate (10^–3^ M) to inhibit the activity of superoxide dismutase. After centrifugation of homogenate at 10,000 rpm on 4 °C for 15 min, the O_2_^•−^ content was measured in the supernatant by its capacity to reduce nitroblue tetrazolium (NBT, 2.5 × 10^−4^ M) with a UV–Vis spectrophotometer (UV-1800, Shimadzu, Kyoto, Japan). The absorbance was taken at 540 nm and the content was expressed as μmol O_2_^•−^g^−1^ fresh weight (FW) of seeds by using the molar absorption coefficient of 12.8 mM^−1^ cm^−1^.

Nitric oxide (NO) content was measured by Greiss reagent (1% sulphanilamide + 0.1% N-1-napthylethylenediamine dihydrochloride in 5% H_2_PO_4_ solution) as described in the method of Zhou et al. [39]. It was estimated as absorbance of the sample at 540 nm with a UV-Vis spectrophotometer (UV-1800, Shimadzu, Japan) using a standard curve prepared with NaNO_2_. The NO content was expressed in nmol NO g^−1^ fresh weight of seeds.

### 2.7. Nitric Oxide Synthase like (NOS-like) and Nitrate Reductase (NR) Activity

NOS-like activity was determined by the method described by Lin et al. [40] after homogenising the sample (1 g) in a buffer containing of 100 mM HEPES-KOH (pH 7.5), 1mM EDTA, 10% glycerol (*v*/*v*), 5 mM Dithiothreitol, 500 μM Phenylmethylsulphonyl fluoride, 0.1% Triton X-100 (*v*/*v*), 1% polyvinylpyrrolidone (*w*/*v*) and 20 μM flavin adenine dinucleotide (FAD). The homogenate was centrifuged at 12,000 rpm for 20 min at 4 °C, and the NOS-like activity was determined in the supernatant according to González et al. [41]. A 200 μL aliquot of the supernatant was used to detect the NOS-like activity in 1 mL of reaction mixture containing 100 mM phosphate buffer (pH 7.0), 1 mM l-Arg, 2 mM MgCl_2_, 0.3 mM CaCl_2_, 4 μM BH_4_, 1 μM FAD, 1 μM flavin mononucleotide (FMN), 0.2 mM DTT, 0.2 mM NADPH. The decrease in absorbance as a result of NADPH consumption was determined at 340 nm for 5 min. NOS-like activity was calculated using the extinction coefficient of NADPH (ε = 6.22 mM^−1^cm^−1^) and expressed as nmol NADPH used g^−1^ fresh weight of seeds.

The NR activity was determined in vivo by following the protocol given by Jaworski [42]. The chopped tissue (250 mg) was mixed in the reaction mixture containing 100 mM phosphate buffer (pH 7.2), 25% isopropanol and 200 mM potassium nitrate and kept undisturbed at 30 °C for 2 h. The nitrite formed in the reaction mixture after incubation was measured at 540 nm following azo-coupling through naphthalenediamine-dihydrochloride and sulphanilamide. The NR activity was represented as nmol NO_2_ g^−1^ FW of seeds h^−1^.

### 2.8. RNA Extraction and Gene Expression Analysis Using Quantitative Real-Time PCR (qPCR)

The soybean seeds (24 h soaked) from unprimed and magnetoprimed seeds were crushed in liquid nitrogen, and total RNA was extracted using RNAiso Plus reagent (TaKaRa, Kusatsu, Japan). The genomic DNA contamination was removed by treating all samples with DNase I (Thermo scientific, Waltham, MA, USA) following the manufacturer’s protocol. The integrity of all RNA samples was confirmed on 1.2% (*w*/*v*) denaturing agarose gel electrophoresis followed by RNA quantification using NanoDrop™ 2000 (Thermo Scientific, Waltham, MA, USA). Following this, the cDNA was synthesized with approximately 2 µg total RNA template and oligo (dT)_18_ primer by using PrimeScript™ 1st strand cDNA Synthesis Kit (TaKaRa Kusatsu, Japan) following manufacturer’ s instructions. 

The qRT-PCR reaction (10 µL) was set using PowerUp^TM^SYBR^TM^green master mix (Applied Biosystem, Waltham, MA, USA) and was performed in StepOnePlus^TM^Real-time PCR system (Applied Biosystem). The amplification of each gene in the PCR reaction was performed using initial denaturation at 95 °C for 10 min, followed by 40 cycles of 95 °C for 10 s, 58 °C for 30 s, and 72 °C for 30 s. Further, dissociation curve analysis was performed from 60 °C to 95 °C with an increment of 0.3 °C/s for data acquisition. The specificity of PCR products amplified from each primer pair was confirmed through dissociation curve analysis. The Elongation factor-1-alpha (*Glyma.19G052400*) and Tubulin alpha-2 (*Glyma.20g27280*) genes were used as internal control (Table 2) and their Ct values were used to normalize the expression of target genes. The relative expression level of genes in fold change with respect to UP samples of control and control + NaCl was calculated using the 2^−^^ΔΔ^^CT^ method [43].

### 2.9. Statistical Analysis

Statistical analysis was performed on Microsoft Excel and Prism 4 (GraphPad Software, LaJolla, CA, USA) software where mean and standard errors (n = 3) were calculated, and the analysis of variance (ANOVA) was followed by post hoc Newman–Keuls Multiple Comparison Test.

## 3. Results

### 3.1. Salt Tolerance Index (STI) 

**Figure 1:**
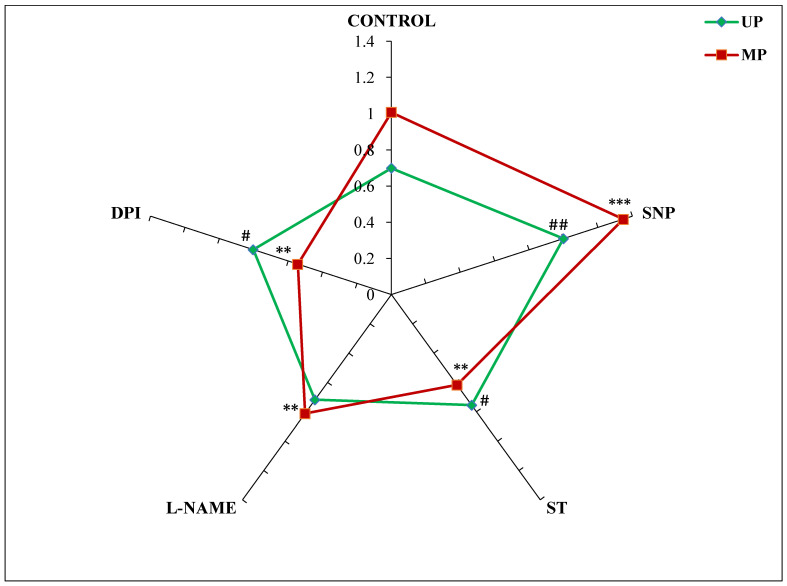
Effect of magnetopriming (200 mT SMF for 1 h) on salt tolerance index (STI) based on dry weight of 72 h germinated soybean seeds grown in different modulators of NO and ROS production under non-saline and saline (50 mM NaCl) conditions. Unprimed = UP, Magnetoprimed = MP. The data are represented as mean ± SE (n = 3). ## *p* < 0.01, # *p* < 0.05 indicates the significant difference between STI of unprimed seeds in the absence (control) versus presence of modulators of NO/ROS production under non-saline or saline conditions; *** *p* < 0.001, ** *p* < 0.01 indicates the significant difference between STI of MP seeds in the absence (control) versus presence of modulators of NO/ROS production under non-saline or saline conditions.

The STI values showed a considerable difference in salt tolerance among the seedlings obtained from magnetoprimed and unprimed seeds germinated in different modulators of NO and ROS. Magnetopriming increased the salt tolerance by 44% in comparison to the UP seeds. The NO donor-SNP also enhanced the STI in the seedlings emerging from UP (43%) and MP (34%) seeds as compared to their respective controls (Figure 1). The NO and ROS inhibitors caused a slight increase (from 2% to 14%) in STI values in seedlings from unprimed seeds. However, these inhibitors significantly reduced the values of magnetopriming-induced salt tolerance index, which was maximally reduced with DPI (45%) followed by ST (39%) and L-NAME (19%) as compared to their magnetoprimed controls (Figure 1).

### 3.2. Effect of Magnetopriming on Na^+^/K^+^ Ratio under Salt Stress

**Figure 2:**
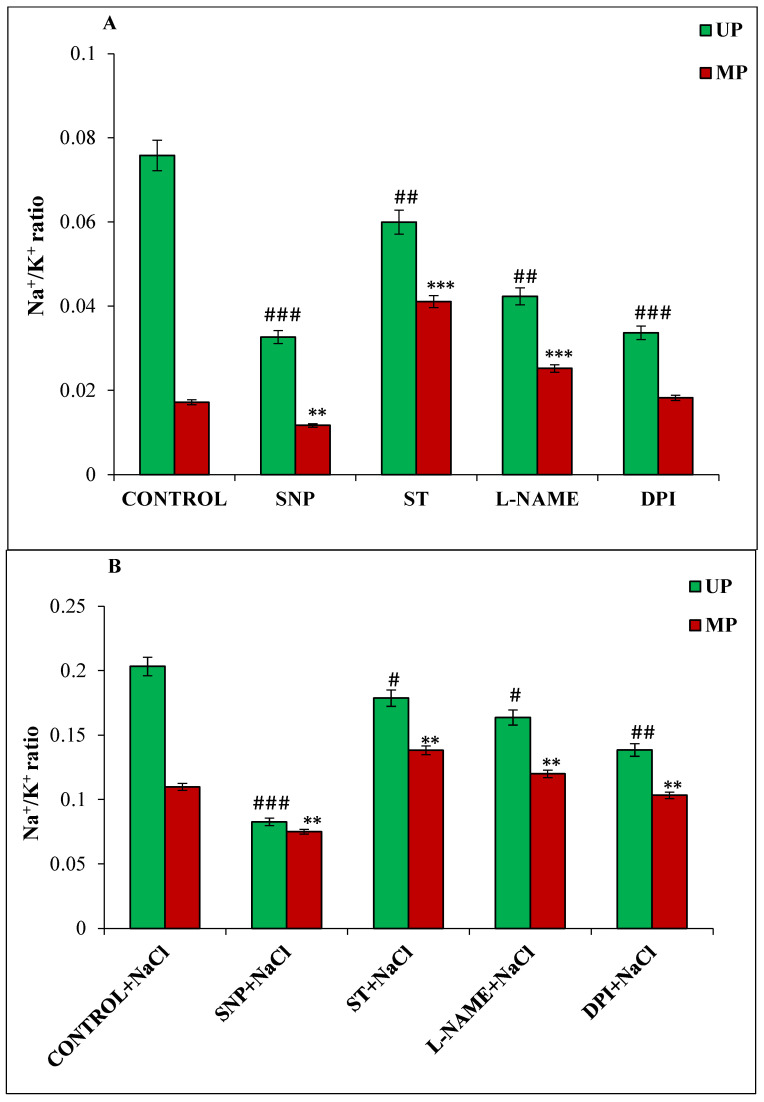
Effect of magnetopriming (200 mT SMF for 1 h) on Na^+^/K^+^ ratio in 72 h germinated soybean seeds in different modulators of NO and ROS production under non-saline (**A**) and saline (**B**) conditions. The data are represented as means ± SE (n = 3). #*## p* < 0.001, *## p* < 0.01, *# p* < 0.05 indicates the significant difference amongst the UP seeds grown in control with the UP seeds grown in different modulators of NO/ROS production under non-saline or saline conditions; *** *p* < 0.001, ** *p* < 0.01 indicates the significant difference amongst the seeds of MP seeds grown in control with the seeds of magnetoprimed seeds grown in different modulators of NO/ROS production under non-saline or saline conditions.

A low cytosolic concentration of Na^+^/K^+^ ratio in tissues growing under salt stress indicates their tolerance potential. In the present study, the Na^+^/K^+^ ratio was less in germinating seedlings from magnetoprimed seeds compared to unprimed seeds under both the growing conditions (Figure 2A,B). SNP-treated seedlings showed 32% lower Na^+^/K^+^ ratio in magnetoprimed seedlings under non-saline and saline conditions as compared to their respective magnetoprimed control (Figure 2A,B). On the other hand, Na^+^/K^+^ ratio was significantly increased in magnetoprimed seedlings of ST (139%) and L-NAME (47%) treatments as compared to their magnetoprimed controls under non-saline conditions (Figure 2A). However, the magnitude of change was less under salinity, as ST treatment showed 26%, and L-NAME 9%, increase in the seedlings obtained from magnetoprimed seeds as compared to their magnetoprimed controls (Figure 2B). A significant difference was also observed in Na^+^/K^+^ ratio under non-saline and saline conditions for unprimed seeds treated with the NO and ROS modulators. 

### 3.3. Effect of Magnetopriming on Phytohormones (ABA, GA and IAA) under Salt Stress

The results of hormones, NO and superoxide content as well as enzyme activities are presented as the relative change in magnetoprimed over unprimed seeds under different modulators in non-saline and saline environments.

**Figure 3:**
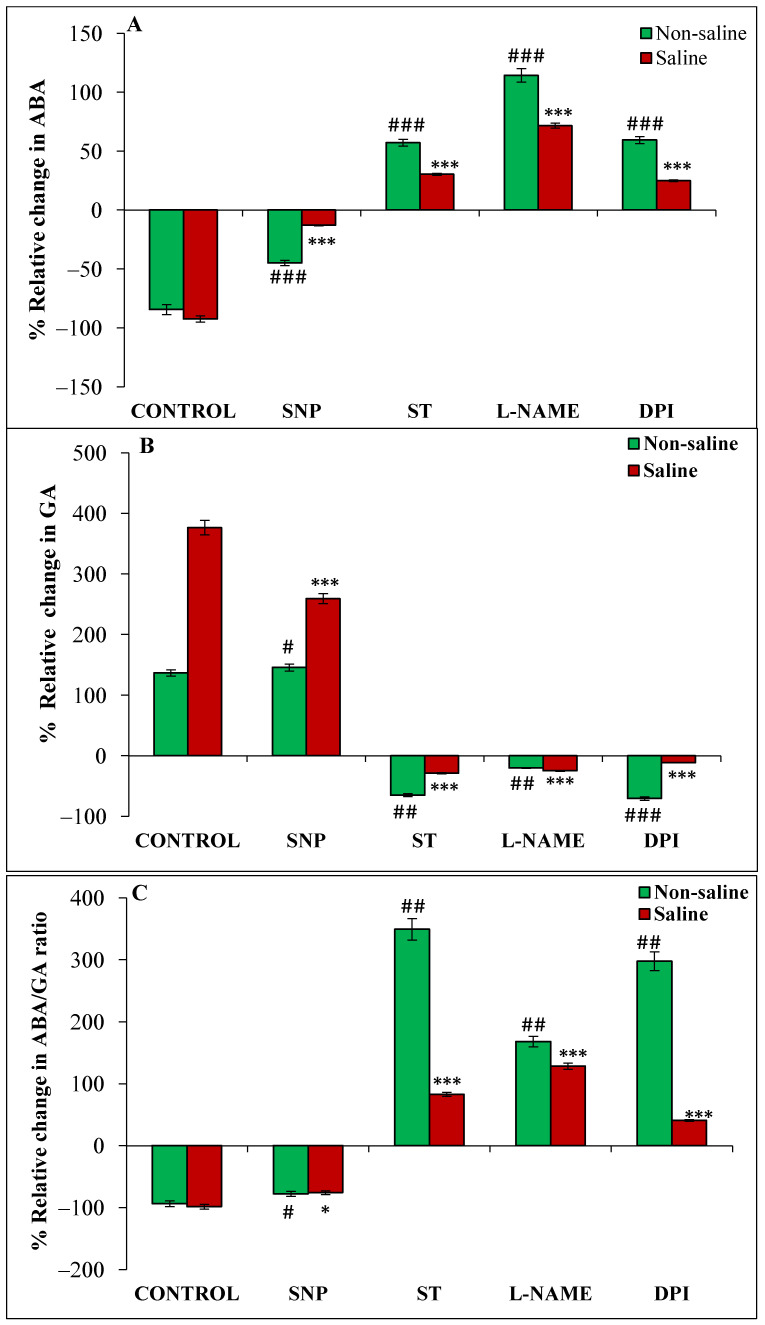

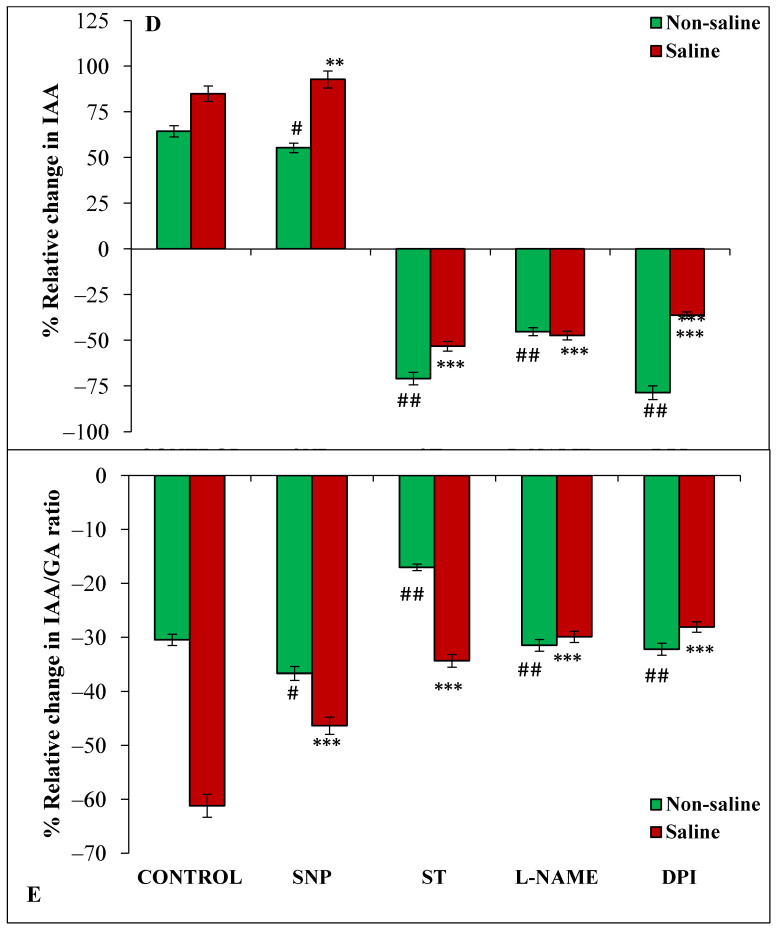
Relative change (%) in ABA (**A**), GA (**B**), ABA/GA (**C**), IAA (**D**) and IAA/GA (**E**) of magnetoprimed over unprimed seeds kept for 24 h in different modulators of NO and ROS production under non-saline and saline conditions. The data are represented as mean values of relative change ± SE (n = 3). ### *p* < 0.001, ## *p* < 0.01, # *p* < 0.05 shows significant difference amongst the relative change in the seeds grown in control with the seeds grown in different modulators of NO/ROS production under non-saline conditions; *** *p* < 0.001, ** *p* < 0.01, * *p* < 0.05 shows significant difference amongst the relative change in the seeds grown in control with the seeds grown in different modulators of NO/ROS production under saline conditions.

Magnetopriming decreased the ABA content in germinating seeds as compared to unprimed ones in non-saline and saline conditions (Figure 3A). On the other hand, GA significantly increased in magnetoprimed seeds under both the experimental conditions; the percentage of increase being more in saline (376%) than non-saline (136%) conditions (Figure 3B). Also, ABA/GA ratio was lower in seedlings from magnetoprimed seeds in both the non-saline (93%) and saline (98%) conditions as compared to the unprimed seeds (Figure 3C). The donor of NO decreased, and inhibitors of NO and ROS increased, the magnetoprimed-induced ABA/GA content in both the conditions as compared to their respective controls (Figure 3C). The relative increase was significantly high (168 to 349%) with NR and ROS inhibitors under non-saline conditions, whereas it was within a range of 41–128% under saline conditions. 

The trend of IAA content under various treatments was similar to that of GA in the germinating seeds. There was an increase of 64% and 85% in magnetoprimed seeds over unprimed seeds in non-saline and saline environments, respectively (Figure 3D). Similarly, SNP (NO donor) also caused a significantly higher percentage of enhancement in IAA content of magnetoprimed seeds, though the extent of promotion (93%) was higher under saline conditions compared to a non-saline medium (55%) (Figure 3D). The inhibitors ST, L-NAME and DPI caused a reduction in magnetoprimed induced IAA content under both the conditions as compared to their respective controls (Figure 3D). Further, we have also observed that IAA/GA ratio was remarkably decreased by magnetopriming in non-saline (30%) and saline (61%) conditions with respect to the unprimed ones (Figure 3E); SNP treatment caused 37% and 46% reduction in IAA/GA ratio in seedlings from magnetoprimed seeds under non-saline and saline conditions, respectively, as compared to the unprimed ones. The IAA/GA ratio was also significantly reduced by magnetopriming in ST, L-NAME and DPI treatments under salt stress, but the extent of reduction was lesser than control and SNP treatment (Figure 3E).

### 3.4. Effect of Magnetopriming on Superoxide (O_2_^•−^), Nitric Oxide (NO) Content, NOS-like and NR Activities under Salt Stress

**Figure 4:**
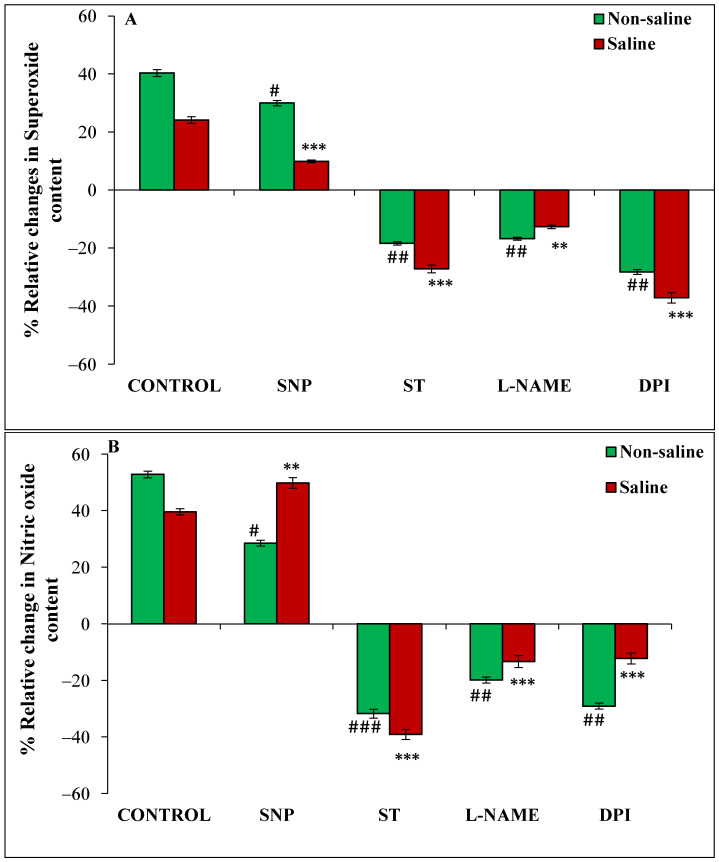
Relative change (%) in superoxide radical (**A**) and nitric oxide(**B**) content of magnetoprimed over unprimed seeds kept for 24 h in different modulators of NO and ROS production under non-saline and saline conditions. The data are represented as mean values of relative change ± SE (n = 3). ### *p* < 0.001, ## *p* < 0.01, # *p* < 0.05 shows significant difference amongst the relative change in the seeds grown in control with the seeds grown in different modulators of NO/ROS production under non-saline conditions; *** *p* < 0.001, ** *p* < 0.01 shows significant difference amongst the relative change in the seeds grown in control with the seeds grown in different modulators of NO/ROS production under saline conditions.

**Figure 5:**
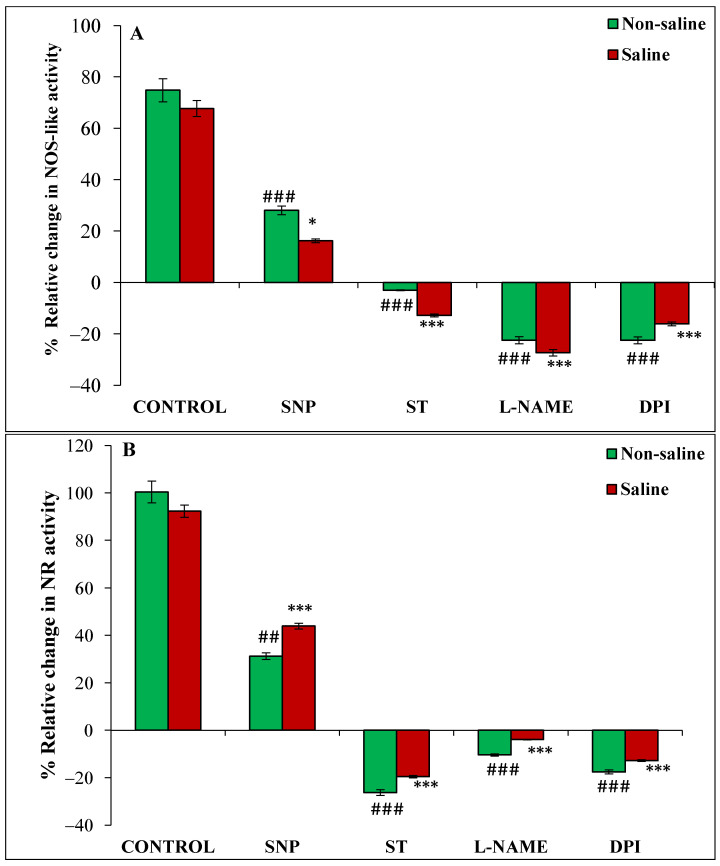
Relative change (%) in NOS-like (**A**) and NR (**B**) activities of magnetoprimed over unprimed seeds kept for 24 h in different modulators of NO and ROS production under non-saline and saline conditions. The data are represented as mean values of relative change ± SE (n = 3). ### *p* < 0.001, ## *p* < 0.01 shows significant difference amongst the relative change in the seeds grown in control with the seeds grown in different modulators of NO/ROS production under non-saline conditions; *** *p* < 0.001, * *p* < 0.05 shows significant difference amongst the relative change in the seeds grown in control with the seeds grown in different modulators of NO/ROS production under saline conditions.

The O_2_^•−^ and NO content and the activities of NOS-like and NR in the 24 h soaked seeds were significantly increased in magnetoprimed seeds under non-saline condition (Figure 4 and Figure 5). The addition of SNP during imbibition resulted in a significant reduction in all observations compared to their respective controls in non-saline environment. However, under salinity the response obtained differed, as a significant increase in NO content was observed by SNP as compared to the control. The inhibitors ST, L-NAME and DPI also showed a significant percentage of reduction in relative values when compared with their controls (Figure 4 and Figure 5A,B).

The magnetoprimed seeds showed 40% and 24% increase in O_2_^•−^content in non-saline and in saline conditions, respectively, over the unprimed seeds (Figure 4A). An increase of 30% and 10% was evident with NO donor treatment, in primed seeds over unprimed ones, under non-saline and saline conditions, respectively (Figure 4A). Imbibition with inhibitors such as DPI led to 28, ST-18 and L-NAME-17% reduction in relative change in primed over unprimed ones under non-saline conditions (Figure 4A). A similar trend was also seen in presence of salt stress as DPI caused a reduction by 37%, ST 27% and L-NAME 13% (Figure 4A).

The relative change in NO content in 24 h imbibed seed was increased by magnetopriming (53% in non-saline and 40% in saline) over the unprimed seeds (Figure 4B). An increase of 28% and 50% was evident with NO donor treatment in primed seeds over unprimed under non-saline and saline conditions (Figure 4B). The lower levels were observed with inhibitors as ST recorded 32%, L-NAME 20% and DPI 29% in magnetoprimed over unprimed ones under non-saline conditions (Figure 4B). A similar trend was observed in the presence of salt stress also; ST caused a reduction by 39%, L-NAME by 13% and DPI by 12% (Figure 4B).

The relative change in the activity of NOS-like enzyme was increased by 75% and 68% in magnetoprimed compared to unprimed seeds in the absence and presence of salt stress, respectively (Figure 5A). SNP also caused an increase in NOS-like activity in the magnetoprimed seeds under non-saline (28%) and saline (16.3%) conditions, but the extent of promotion was less than their respective controls (Figure 5A). Magnetopriming-induced NOS-like activity was reduced by ST, L-NAME and DPI in both non-saline and saline conditions; the maximum reduction was recorded under L-NAME treatment in non-saline (22%) and saline (27%) conditions (Figure 5A).

Magnetopriming caused marked enhancement in the NR of imbibed soybean seeds under non-saline (100%) and saline (92%) conditions (Figure 5B). The NO donor SNP caused 31% and 44% increase in NR activity in seedlings from primed seeds as compared to their unprimed ones under non-saline and saline conditions, respectively (Figure 5B). The relative increase in NR was higher than NOS-like activity of magnetoprimed seeds treated with SNP under saline conditions (Figure 5A,B). All the inhibitors of NO/ROS production reduced the NR activity in both saline and non-saline conditions, but maximally in ST treatment (26% in non-saline and 20% in saline conditions) (Figure 5B).

### 3.5. Identification of Putative Seed Specific GmNOS-like and GmNR Genes in Soybean Genome

NO has been shown to be an important signalling molecule performing various physiological functions in plants. NO synthesis from oxidative and reductive routes is evident in various plant species [44]. In model plant *Arabidopsis thaliana*, there are two nitrate reductase genes, named *AtNIA1* (*AT1G77760*) and *AtNIA2* (*AT1G37130*) [44,45]. Since there is no information available for genes encoding for nitrate reductase genes (*GmNR*) in soybean genome, we searched for the orthologs of Arabidopsis *AtNIA1* and *AtNIA2* genes in the soybean genome using the PANTHER tool (http://www.pantherdb.org/) accessed on 9 December 2019 and identified a total of five putative nitrate reductase encoding genes in the soybean genome (Supplementary Table S1). The presence of nitric oxide synthase genes in higher plants is controversial, but NO production via oxidative route (NOS-like activity), supported by several reports, could not be ignored [45]. Therefore, to find the putative *NOS-like* genes in soybean, the Arabidopsis gene *AtNO1/AtNOS1* (*AT3G47450*) was used, and two *NOS-like* genes were identified in soybean (Supplementary Table S1). Since we were interested to select the putative *GmNR* and *GmNOS-like* genes expressing in the seeds or early germinating seedlings, we checked the putative seed-specific expression profile of identified soybean *GmNR* and *GmNOS-like* genes on Phytozome 13 (https://phytozome-next.jgi.doe.gov/, accessed on 30 May 2022). On the basis of putative expression profile, we selected two *GmNOS-like* (*Glyma.09G224600*; *GmNOS-like 1* and *Glyma.12G012400*; *GmNOS-like 2*) and *GmNR* (*Glyma.13G084000*; *GmNR1* and *Glyma.06G109200*; *GmNR2*) genes (Supplementary Table S1). 

### 3.6. Effect of Magnetopriming on the Expression of Genes GmNOS-like and GmNR Involved in NO Synthesis

**Figure 6:**
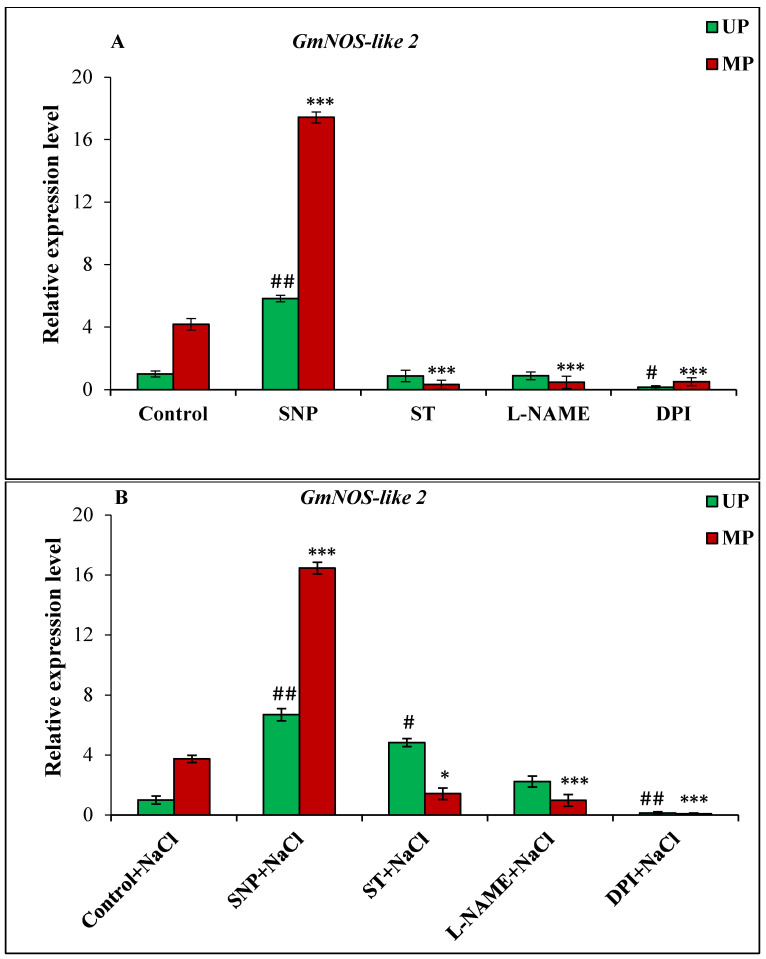
Relative expression analysis of *GmNOS-like 2* gene in soybean seeds kept for 24 h in different modulators of NO and ROS production under non-saline (**A**) and saline (**B**) conditions. The data are represented as the means ± SE (n = 3). The fold change in the expression values was calculated using the 2^−ΔΔCT^ method given by Livak and Schmittgen [43]. The alpha-tubulin (*Glyma.19G052400*) gene was used as internal control to normalize the expression, and control UP sample was used as a calibrator in both non-saline and saline treatments. *## p* < 0.01, *# p* < 0.05 indicates the significant difference amongst the UP seeds grown in control with the UP seeds grown in different modulators of NO/ROS under non-saline or saline conditions; *** *p* < 0.001, * p < 0.05 indicates the significant difference amongst MP seeds grown in control with the MP seeds grown in different modulators of NO/ROS under non-saline or saline conditions.

The *GmNOS-like 1*, *GmNOS-like 2*, *GmNR1* and *GmNR2* genes were selected to check their expression in the unprimed/primed germinating seeds under saline and non-saline conditions. However, primers used to detect *GmNOS-like 1* and *GmNR2* genes could not generate any amplicon in the PCR reaction, and hence no data are provided. For all the genes, relative expression was calculated with expression of germinating unprimed seeds under non-saline and saline conditions as respective calibrators under both the conditions. Under non-saline conditions, *GmNOS-like 2* gene from magnetoprimed seedlings showed 4.18-fold higher expression over the seedlings from unprimed seeds (Figure 6A). The addition of NO donor increased the expression by 5.83-fold in unprimed seedlings and 17.42-fold in the magnetoprimed ones (Figure 6A). The treatment with ST, L-NAME, and DPI severely inhibited the expression of *GmNOS2* gene in the magnetoprimed seedlings (Figure 6A).

The magnetoprimed seedlings showed significantly up-regulated *GmNOS-like 2* expression by 3.74-fold in NaCl treatment compared to the unprimed seedlings (Figure 6A). In addition, SNP treatment under salinity raised the *GmNOS2* expression level by 6.69-fold in the unprimed and 16.46-fold in magnetoprimed seeds (Figure 6B). The results with inhibitors varied under non-saline and saline conditions with ST treatment showed 4.83-fold increase, and with L-NAME 2.23-fold increase, in unprimed ones under salinity, whereas others showed significantly decreased *GmNOS-like 2* expressions in both conditions (Figure 6B). 

**Figure 7:**
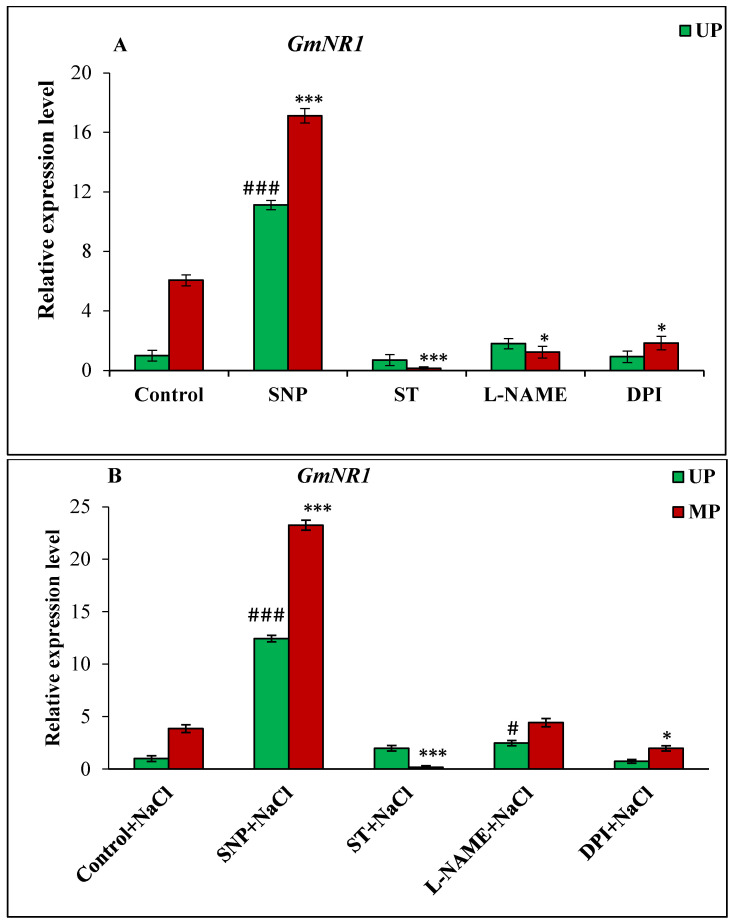
Relative expression analysis of *GmNR1* genes in soybean seeds kept for 24 h in different modulators of NO and ROS production under non-saline (**A**) and saline (**B**) conditions. The data are represented as the means ± SE (n = 3). *### p* < 0.01, *# p* < 0.05 indicates the significant difference amongst the UP seeds grown in control with the UP seeds grown in different modulators of NO/ROS under non-saline or saline conditions; *** p < 0.001, * p < 0.05 indicates the significant difference amongst the MP seeds grown in control with the MP seeds grown in different modulators of NO/ROS under non-saline or saline conditions.

In the case of nitrate reductase genes, the *GmNR1* gene was up-regulated by 6.06-fold in the seedlings of magnetoprimed seeds over seedlings of unprimed seeds under non-saline conditions (Figure 7A). The addition of SNP as NO donor enhanced the transcript level of *GmNR1* gene in both unprimed (11.12-fold) and magnetoprimed (17.12-fold) seedlings compared to unprimed controls. The treatment of magnetoprimed seeds with ST, L-NAME and DPI inhibitors significantly reduced the *GmNR1* transcript level under the non-saline conditions (Figure 7A).

The enhanced transcription of *GmNR1* gene by 3.86-fold in magnetoprimed seedlings over their unprimed controls was observed under salinity (Figure 7B). The NO donor changed the expression of *GmNR1* in both unprimed (12.43-fold higher) and magnetoprimed (23.26-fold) seedlings with respect to the unprimed calibrator under saline environment (Figure 7B). The ST inhibitor of NO caused remarkable reduction in *GmNR1* expression in the magnetoprimed seedlings (Figure 7B). 

## 4. Discussion

A reduced percentage of germination and poor seedling growth in soybean under salinity is the combined outcome of cell water relation and specific ion toxicity [6,46,47]. Seed priming helps to activate the plant immune system through a network of signalling molecules, phytohormones and stress-responsive proteins that empower them to safeguard against abiotic and biotic stresses [15,34,48,49]. Magnetopriming of seeds has been shown to effectively help the seeds to withstand stress effects during the germination process in various crops [9,12,14]. Previously, Kataria et al. [15] found that seed germination was enhanced by 23% via MP of soybean seeds under salt (50 mM NaCl) stress. Our study revealed that magnetopriming increased the stress tolerance index (STI) in the primed soybean seeds under 50 mM NaCl salinity. The improvement in STI with NO donor (SNP) and its loss with NO/ROS inhibitors ST, L-NAME and DPI in both unprimed and primed seeds indicated the direct involvement of NO along with ROS towards imparting salt tolerance. The negative effect of salinity on seed germination and plant growth was observed to be reduced by the external application of SNP in *Chenopodium* and *Raphanus* [50,51].

The salinity tolerance in plants is maintained by a low Na^+^/K^+^ ratio [52] which is mainly disturbed by high cellular concentrations of Na^+^ [53]. Magnetopriming restricted the influx of Na^+^ in the germinating seeds, as the seedlings showed lower ratios under saline condition. Likewise, in an earlier study, magnetopriming of wheat seeds helped in maintaining a lower Na^+^/K^+^ ratio under salinity [54]. When we treated the seeds with SNP it favoured a further reduction in both unprimed and primed seeds. In addition, the exogenous NO treatment improved salt tolerance by maintaining low levels of Na^+^ in Jatropha seedlings [55] and enhanced production of ascorbate, glutathione, total phenolic content, proline and flavonoids in spinach [56] and tomato [57]. Also, the SNP pre-treatment significantly increased the K^+^ and decreased Na^+^ contents, consequentially increasing the level of K^+^/Na^+^ ratio in *Brassica chinensis* leading to improved germination and seedling growth under NaCl stress [24]. Molecular analysis of NO-treated plants growing under salt stress enhanced the expression of *SOS1* and *NHX1* [58] and *H^+^-ATPase* and *H^+^-PPase* [59] genes in the stressed leaves which were suggested to reduce Na accumulation and increase the activity of Na^+^/H^+^ exchange in the tissues, respectively.

Endogenous NO content increased in the magnetoprimed seeds in the absence or presence of NO donor, whereas NO inhibitors (ST and L-NAME) showed a reduction in content under both the non-saline and saline growing conditions. NO is a redox signalling molecule that controls various physiological processes in the plants such as germination, flowering, senescence and multiple stresses [2,22]. Under stress conditions, external application of NO alleviates the plant by upregulating its antioxidant defence system and helping in ROS homeostasis [60,61]. ROS homeostasis has been linked to an enhanced rate of germination and vigor in magnetoprimed seeds of soybean [62]. Amongst the various mechanisms involved in buffering the levels of ROS, NO is a proven candidate [63,64]. In our experiments, the treatment with DPI (ROS inhibitor) showed low levels of NO, indicating that NO may not be required for the scavenging process as ROS production is inhibited. Thus, the ability of DPI to restrict the production of ROS as well as NO, and the inability of NO-modulators to prevent the rise in ROS levels, indicated that inhibition of ROS production controlled the synthesis of NO in the seedlings. Similarly, Shan and Yang [65] also found that DPI reduced the NO production along with ROS in *Agropyron cristatum*. Both ROS- and NO-mediated adaptation to stress is brought about by these molecules acting as retrograde signals in the cells to affect the function of target proteins through redox or S-nitrosylation post-translational modifications [66].

The two major pathways of NO synthesis in the plants are (i) oxidative-oxidation of L-arginine by NOS-like enzyme and (ii) reductive-reduction of nitrate by nitrate reductase (NR). In the plants, measurement of NOS-like activity by employing NOS inhibitors like L-NAME have suggested the existence of arginine-dependent NO production pathway [44,67]. Our analysis of the activity of both the enzymes showed a similar trend between NR activity and NO production in all the treatments except ST under non-saline and saline environments. On the other hand, NOS-like activity showed a higher reduction in relative values of magnetoprimed over unprimed in L-NAME treatment, but the corresponding reduction in NO content was comparatively less in this treatment. Higher reduction in NO content could be positively correlated with the reduced values of NR activity under ST treatment. The relative expression of the genes coding for NR and NOS-like activities confirmed and supported our results, as the gene *GmNR1* recorded a higher (23.26 fold) abundance of transcripts in exogenous SNP in comparison to *GmNOS-like 2* under the same treatment under saline conditions. Additionally, the results of the present study strengthen the conclusion of our previous study that confirmed the involvement of NR (through spectrophotometric and florescent microscopic dye) as the major contributor to NO production in magnetoprimed soybean seedlings exposed to salinity stress [15].

The preference of a reductive over an oxidative pathway in the germinating seeds may be the result of excess formation of NAD(P)H in respiration which increases during the germination process [68,69]. Under chilling stress, the higher rate of respiration and ATP production stimulated the growth of seedlings obtained from primed rice seeds [69,70]. In the reductive pathway, nitrate is reduced to nitrite by utilization of cytosolic NAD(P)H followed by its further reduction to NO by electron transfer from NAD(P)H to nitrite [71]. Magnetopriming and SNP supplementation both increased the *GmNOS-like 2* and *GmNR1* expression which supports the concomitant increment in NO production, although the transcript abundance of *GmNR1* was more than that of *GmNOS-like 2* gene. We also observed that ST, L-NAME and DPI caused the downregulation in gene expression of both *GmNOS-like 2* and *GmNR1* under both the conditions. However, the ST treatment caused the maximum reduction in *GmNR1* transcript level as compared to L-NAME, which caused reduction in *GmNOS-like 2* transcript levels in the present study.

In plants, the NO/ROS regulate various metabolic processes through their control on the perception, metabolism or signal transduction of different hormones like ABA, auxins, gibberellin, cytokinins and ethylene [72,73]. NO governs the ABA signalling pathway at different steps involving the S-nitrosylation of PYR/PYL/RCAR complex or SUCROSE NON-FERMENTING (SNF)-RELATED PROTEIN KINASE2.6 (SnRK2) to inactivate them or cause proteasomal degradation of ABSCISIC ACID INSENSITIVE5 to break seed dormancy and promote germination [74]. Unlike ABA, GA is known to promote seed germination in various crops [30,75,76]. Therefore, in our previous study, we elucidated that ABA does not exclusively control the germination process, but the hormonal homeostasis under high ROS levels in magnetoprimed tomato seeds is managed by the ABA/GA ratio to enhance the rate of germination under unstressed conditions [48], which was also reported in the previous studies [30,77,78,79]. We have also recorded a significant increase (301%) in ABA content in UP seeds grown in saline conditions as compared to UP seeds of non-saline conditions. In addition, there was a 200% increase in MP seeds grown in saline conditions as compared to MP seeds grown in non-saline conditions (data not shown). The lesser increase in ABA by MP in saline conditions is due to higher tolerance of MP seeds towards salinity stress. The levels of both the phytohormones ABA and GA were significantly altered during seed germination even under salt-stressed conditions with and without the participation of NO [72,80,81,82]. In the current study, we also observed that NO exercised its control on ABA/GA, as the relative change in the ratio of ABA/GA in magnetoprimed vs. unprimed seeds reduced in absence and presence of the NO donor, but the inhibitors showed a positive change in ABA/GA under both non-saline and saline conditions. Similarly, ROS produced by NADPH oxidase enzyme in barley seeds, induced GA biosynthesis in the embryo which further activated alpha-amylase enzyme in the aleurone layer [83] to hydrolyse starch for seedling growth.

In our experiments, the involvement of IAA in the NO-induced tolerance to salinity at germination stage indicates the crosstalk of auxins with the signalling molecule (NO). IAA levels increased in magnetoprimed seeds as compared to unprimed seeds in absence and presence of NO donor treatment, both under non-saline and saline conditions. An increase in IAA content along with GA was reported in magnetoprimed faba bean [84], which could be a result of hydrolysis of amide-IAA conjugates stored in the mature seed [34,85]. We also found that the trend in relative change of IAA was similar to that of GA under all the treatments. We derived the ratio of IAA/GA and noticed that the ratios were severely reduced in control and SNP treatments by magnetopriming under saline conditions. Lower ratios of IAA/GA are favourable for germination under both the growing environments, as higher IAA levels in the germinating seeds, if not checked, can inhibit seed germination in an ABA dependent manner [31]. Liu et al. [31] showed that transgenics over-expressing auxin (iaaM-OX) stopped the germination of Arabidopsis seeds, while mutants with low levels of auxins improved germination rate. Belin et al. [86] identified ABI3 as a pivotal regulatory component for the maintenance of auxin-associated dormancy. Recently, Hussain et al. [33] found that the auxin signalling repressor IAA8 accumulated during germination and down regulated the expression of ABSCISIC ACID INSENSITIVE3 (ABI3), which is a negative regulator of germination in *Arabidopsis thaliana* leading to the enhancement in germination rate. Although we have observed an increase in IAA levels in the magnetoprimed seeds, which may be the released product from IAA conjugate breakdown, the stimulation in germination was not hampered, as the ratio of IAA/GA was low in all the treatments. Our study suggested that magnetopriming produces NO that regulates the homeostatic control over the hormones (ABA, GA and IAA) and reduces the influx of Na^+^ in the germinating seed, thus inducing salt tolerance in soybean.

## 5. Conclusions

**Figure 8:**
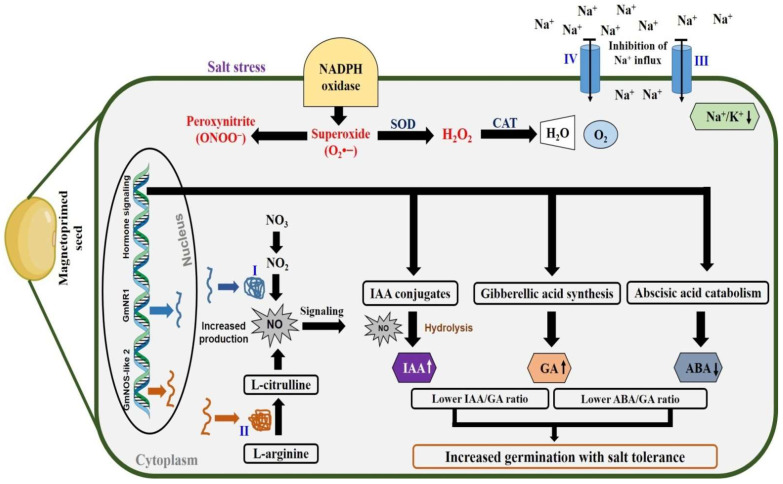
Proposed model for magnetopriming-induced salt tolerance in soybean seeds. Magnetopriming of soybean seeds promotes NO synthesis via (I) Nitrate reductase and (II) Nitric oxide synthase-like. There may be involvement of (III) Plasma membrane H^+^-ATPase and (IV) Na^+^/H^+^ antiporter. The Na^+^ influx may be regulated by enzyme activities of plasma membrane III and IV. NO regulates homeostatic control of ABA/GA and IAA/GA ratio in magnetoprimed seeds. Overall, magnetopriming-induced NO-mediated homeostasis of germination hormones and lower Na^+^/K^+^ ratio improve the salt tolerance index in soybean seeds. SOD = Superoxide dismutase, CAT = Catalase.

Our study is summarised in a schematic pathway (Figure 8) which illustrates that magnetopriming of soybean seeds enhanced the NO production by up-regulation of genes of enzymes related to NOS-like and NR along with their enhanced activities. The comparison of the two biosynthetic pathways showed that the contribution of NR towards NO production is more than that of NOS-like, which is supported by the results obtained from the treatment of different inhibitors (ST and L-NAME) of the two enzymes under non-saline and saline conditions. The *GmNOS-like 2* and *GmNR1* genes could be ideal candidates to be characterised towards their involvement in the NO production in soybean. There is an inverse correlation between STI and Na^+^/K^+^ ratio, i.e., the STI values are higher and Na^+^/K^+^ ratio lower in the seedlings that emerged from MP and SNP + MP treatments in non-saline as well as in saline conditions. The higher GA and IAA content with lower ABA/GA and IAA/GA ratios in the magnetoprimed seeds under both the non-saline and saline conditions indicated that priming induced NO production had lowered the Na^+^/K^+^ ratio to increase the salt tolerance index in soybean seeds. Thus, MP-induced NO production is directed towards the homeostasis of these hormones to favour the germination and seedling growth in the presence or absence of salt.

## Figures and Tables

**Table 1:** The details of treatments used in present study.

S. No.	Treatment	Description
1.	Control	Unprimed and magnetoprimed seeds kept in distilled water under non-saline condition
2.	Control + NaCl	Unprimed and magnetoprimed seeds kept in 50 mM NaCl for saline condition
3.	ST, L-NAME, and DPI	Unprimed and magnetoprimed seeds kept in NO (SNP, ST and L-NAME) and ROS (DPI) modulators under non-saline condition
4.	ST + NaCl, L-NAME + NaCl, and DPI + NaCl	Unprimed and magnetoprimed seeds kept in NO (SNP, ST and L-NAME) and ROS (DPI) modulators under saline condition

**Table 2:** Primers used in the present study.

S. No.	Gene Locus	Primer Name	Primer Sequence (5′ to 3′)
1.	*Glyma.09G224600 (GmNOS-like 1)*	GmNOS1-F	AATAAGAAGAAAAAGAAGAAA
		GmNOS1-R	TTCGAAGCTGGTGGTGTTTCT
2.	*Glyma.12G012400 (GmNOS-like 2)*	GmNOS2-F	TGTGGACAGTTATGATCCCAA
		GmNOS2-R	AACAGCCTTGGGGACGTGCAC
3.	*Glyma.13G083800 (GmNR1)*	GmNR1-F	GACCGGTTCAAGCTATGGTAC
		GmNR1-R	TTTCTCCAAATTAGGCTGCAC
4.	*Glyma.06G109200 (GmNR2)*	GmNR2-F	GCTACCCCAGCCGCCGCCGCC
		GmNR2-R	AAACGGACAAGGGAAGAGTTC
5.	*Glyma.19G052400 (GmEF1A)*	GmEF1A-F	TGAAGCTGGTATTTCTAAGGA
		GmEF1A-R	GTAACCAACCTTCTTCAAGTAG
6.	*Glyma.20G136000 (GmTUA4)*	GmTUA4-F	CGTGCAGTGTTTGTAGATCTT
		GmTUA4-R	GATCAACAATCTCTTTCCCAA

## Data Availability

Data can be obtained on request from the authors.

## Supplementary Materials

The following supporting information can be downloaded at: https://www.mdpi.com/article/10.3390/cells11142174/s1, Table S1: List of putative NOS-like and NR genes in soybean. References [45,87] are cited in Supplementary Materials.
