# Supplementary material for: Magnetopriming Actuates Nitric Oxide Synthesis to Regulate Phytohormones for Improving Germination of Soybean Seeds under Salt Stress
Source: Cells. 2022 Jul 12;11(14):2174. doi: 10.3390/cells11142174 (PMC9322440; doi:10.3390/cells11142174)
Supplement: Supplementary file 1 [file cells-11-02174-s001.zip › cells-1776483-supplementary.pdf]

Supplementary Table S1: List of putative NOS-like and NR genes in soybean

S. No.	Gene	Arabidopsis gene	Ortholog in Soybean	Reference
**1.**	Nitrate reductase	*AT1G77760 (AtNIA1)*	Glyma.06G109200 (*GmNR2*)	[45]
			Glyma.13G083800	
			Glyma.13G084000	
			Glyma.14G164900	
			Glyma.14G165000	
		AT1G37130 (AtNIA2)	Glyma.06G109200	[45]
			Glyma.13G084000 (*GmNR1*)	
			Glyma.14G164900	
			Glyma.14G165000	
**2.**	Nitric oxide synthase 1(*AtNOS1*) or Nitric oxide associated 1 (*AtNO1*)	AT3G47450 (*AtNOA1*)	Glyma.09G224600 (*GmNOS-like 1*)	[87]
			Glyma.12G012400 (*GmNOS-like 2*)	
